# Xylose Metabolization by a *Saccharomyces cerevisiae* Strain Isolated in Colombia

**DOI:** 10.1007/s12088-023-01054-z

**Published:** 2023-02-18

**Authors:** Margareth Andrea Patiño Lagos, Jorge Alejandro Cristancho Caviativa, Diana Carolina Tusso Pinzón, Diego Hernando Romero Roa, Thiago Olitta Basso, Mario Enrique Velásquez Lozano

**Affiliations:** 1 Facultad de Ciencias, Instituto de Biotecnología, Universidad Nacional de Colombia – Sede Bogotá, Calle 44 # 45-67 Bloque B5, oficina 703, Bogotá, Colombia; 2 Grupo de Investigación en Procesos Químicos y Bioquímicos, Departamento de Ingeniería Química y Ambiental, Facultad de Ingeniería, Universidad Nacional de Colombia, Bogotá, Colombia; 3 Departamento de Ingeniería Eléctrica y Electrónica, Universidad Nacional de Colombia, Bogotá, Colombia; 4 Departament of Chemical Engineering, University of Sao Paulo, São Paulo, Brazil

**Keywords:** *Saccharomyces cerevisiae*, Xylose, Pentose, Xylitol

## Abstract

*Saccharomyces cerevisiae* (*S. cerevisiae*) is the most widely used yeast in biotechnology in the world because its well-known metabolism and physiology as well as its recognized ability to ferment sugars such as hexoses. However, it does not metabolize pentoses such as arabinose and xylose, which are present in lignocellulosic biomass. Lignocellulose is a widely available raw material, with xylose content of approximately 35% of total sugars. This xylose fraction could be used to obtain high added-value chemical products such as xylitol. One of these yeasts isolated from a Colombian locality, designated as 202-3, showed interesting properties. 202-3 was identified through different approaches as a strain of *S. cerevisiae*, with an interesting consumption of xylose metabolizing into xylitol, in addition with excellent ability as a hexose fermenter with high ethanol yields and shows resistance to inhibitors present in lignocellulosic hydrolysates. The xylose metabolization by the 202-3 strain and their kinetics parameters had not been previously reported for any other natural strain of *S. cerevisiae*. These results suggest the great potential of natural strains for obtaining high value-added chemical products using sugars available in lignocellulosic biomass.

**Supplementary Information:**

The online version contains supplementary material available at 10.1007/s12088-023-01054-z.

## Introduction

There is a wide field of research in yeast strains for the complete use of the sugars available in lignocellulosic biomass and therefore, with the ability to ferment pentoses. Achieving this target in the yeast performance would allow obtaining high added-value products such as alcohols, enzymes and other fine chemicals [1]. Agricultural, municipal, and industrial wastes have the potential to generate biofuels and biopolymers through fermentation processes [2]. Lignocellulosic materials are an abundant and renewable source of sugars, very often available from agricultural residues, with high potential to be used as a substrate for the production of alcohols such as xylitol and ethanol. The research group “*Procesos Químicos y Bioquímicos*” of Universidad Nacional de Colombia (Research group on Chemical and Biochemical Processes) developed the project “Isolation, characterization, and evaluation of native strains from municipality of Puerto López, Meta-Colombia for ethanol production”. To accomplish this work, the group isolated several yeast strains, many of them with interesting characteristics. Particularly, a remarkable strain named as 202-3, that was isolated from 6-month stored molasse from a sugarcane distillery in a rainy season, showed xylose consumption in lignocellulosic hydrolysates. Previously, Cifuentes’ showed an initial sequencing of this 202-3 strain and characterized its genome [3], proposing a possible association of some of its genes with the consumption of xylose.

It is generally accepted that *Saccharomyces cerevisiae* (*S. cerevisiae*) is unable to assimilate xylose [4]. However, some strains of *S. cerevisiae* have shown the ability to consume xylose and produce xylitol. In the study of Gong [5], nine (9) strains of *S. cerevisiae* showed xylose consumption and xylitol production. The one with the highest consumption under aerobic conditions was the ATCC 26497 strain. Similarly, in the study by Hsiao [6] the strain ATCC 23860 consumed a small portion of D-xylose in presence of a mixture of D-xylose, D-xylitol, and D-xylulose. In Van Zyl and colleagues [7], *S. cerevisiae* strains ATCC 26602 and ATCC 26603 showed consumptions of 7.4 gL^− 1^ and 3.1 gL^− 1^ of xylose, respectively, for 6 days in a substrate mixture containing 10 gL^− 1^ of D-xylose, other sugars and components, where the xylose was not utilized in absence of the other sugars. *S. cerevisiae* captures xylose by facilitated diffusion carried only by hexose permeases with low affinity for pentose. Xylose is converted by two sequential reactions into xylulose. Xylose to xylitol by the enzyme xylose reductase (*XR*, encoded by *XYL1*) and xylitol into xylulose is catalyzed by the enzyme xylitol dehydrogenase (*XDH*, encoded by *XYL2*), xylulose is then phosphorylated at the C5-OH position by xylulose kinase (*XK*, encoded by *XYL3*) to xylulose-5-phosphate (X5P) which follows the pentose phosphate pathway [4]. This yeast cannot efficiently metabolize pentoses such as xylose present in hemicellulose hydrolysates because possesses low expression of genes coding for the enzymes *XR*, *XDH* and *XK*.

In this work, the consumption of xylose as the only carbon source by the strain 202-3 and its phenotypic profile are presented. The *S. cerevisiae* species of this strain was confirmed by three approaches: 1—morphological observation through optical and scanning microscopy (SEM), 2—an analysis using species-specific primers that allow identifying species of the *Saccharomyces* clade, and 3—sequencing of their ITS region that showed similarity to the reported sequences of *S. cerevisiae* in the NCBI GenBank database greater than 99 %. The phenotypic profile of strain 202-3 was analyzed using growing and fermentations on xylose. The outcomes of these procedures confirm that strain 202-3 is a *S. cerevisiae* with a modest but appreciable xylose consumption and xylitol production, which make it a very interesting candidate for genetic improvement, even more considering that this is not a common characteristic of this yeast lineage.

## Materials and Methods

### Strains, Medium, and Yeast Cultivation Conditions.

The target strain 202-3 was taken from our strain collection of the Bank of gene and strains of the Biotechnology Institute of Universidad Nacional de Colombia, code IBUN 090-03602 under certification IB-159-17. The following media were used for growth analysis: 1- the synthetic medium YNB (from Yeast Nitrogen Base Without Amino Acids) Y0626-SIGMA-ALDRICH, 2- the fully defined medium referred as “Verduyn medium”, and 3- the complete complex medium YP (Yeast Extract Peptone). Depending on the analyses, these media were enriched with glucose or xylose in several concentrations. YP was referred to as YPD medium (Yeast Extract Peptone Dextrose) when it was enriched with glucose, YPX when it was enriched with xylose, and YPDX when it was enriched with glucose and xylose.

### Yeast Genomic DNA Extraction

For the extraction of yeast genomic DNA, protocols described were applied and the commercial YeaStar™ Genomic DNA Kit (Zymo Research) was used following the manufacturer’s instructions.

### Sequencing DNA of ITS Region

The ITS1-5.8 S-ITS4 region was amplified by PCR from genomic DNA cells using primers ITS1 and ITS4. The products obtained were sequenced by Macrogen Inc. in South Korea through CorpoGen Colombia, using the chemistry of the ABI PRISM® BigDyeTM Terminator Cycle Sequencing kit, capillary electrophoresis, and an ABI PRISM® 3730XL Analyzer (96 capillary type).

### Growth and fermentation.

Fermentations were performed in media with different concentrations of glucose, xylose, or glucose+xylose at 29 °C ± 1 °C. Aliquots of the medium were taken for analysis of sugar consumption, ethanol production and other products. In some characterization tests of strain 202-3, screw-capped flasks with a capacity of 100 mL containing 20 mL of medium were used. Screw cap flasks with an airlock system were also used to prevent the entry of air and facilitate the evacuation of gases, thus generating an anaerobic condition.

Different volume configurations and Erlenmeyer flasks were used for trials to differentiate between aerobic, microaerobic, and anaerobic conditions in Verduyn medium. In the aerobic condition, the Erlenmeyers were 500 mL or 100 mL capacity with inward-faced baffles to increase turbulence agitation of the liquid and promote the gas exchange between the liquid and gaseous phases, allowing a greater oxygen supply. In the microaerobic condition, classic Erlenmeyers flasks of 500 mL and 100 mL were used. In the anaerobic condition, 500 mL Erlenmeyer with a medium volume of 300 mL were coupled to a system with air inlet blocking. The pre-inoculum was performed using 20 gL^− 1^ of xylose as the carbon source. The hydrolysates from crude sugarcane bagasse were tested in 50 mL screw-capped bottles with airlock system, containing 40 mL of medium. These hydrolysates were obtained as follows: (1) Soaking, in sulfuric acid (H_2_SO_4_) 2 % (w/w) at 60 °C for 1 h; (2) Pressing, for reducing acid content until reach 30 %; (3) Pressurized heating, in Parr reactor for 10 min at 160 °C; (4) Neutralization, in ammonium hydroxide (NH_4_OH) 14 % (w/w); and (5) Enzymatic hydrolysis that was realized with Cellic® CTec3 (Novozymes) at 50 °C. The compositions of hydrolysates were for Hydrolysate-a: glucose 15 gL^− 1^, xylose 6 gL^− 1^, arabinose 1.2 gL^− 1^, acetic acid 3 gL^− 1^, and hydroxymethylfurfural 0.05 gL^− 1^; and for Hydrolysate-b: glucose 64 gL^− 1^, xylose 33 gL^1^, arabinose 3.7 gL^1^, acetic acid 7.5 gL^1^, hydroxymethylfurfural 0.2 gL^1^ and furfural 0.9 gL^− 1^ [8]. Plots of growths and fermentations in function of time were obtained.

### Fermentation Parameter Evaluation and Kinetic Analysis

The main quantitative physiological parameters were experimentally determined: biomass yield (Y_X/S_ in gg^− 1^), ethanol yield (Y_Etha/S_ in gg^− 1^), glycerol yield (Y_Gly/S_ in gg^− 1^), xylitol yield (Y_Xyl_ in gg^− 1^,), ethanol volumetric productivity (Qp in gL^1^h^1^), and D-xylose or glucose consumption (in gL^− 1^).

### HPLC Quantitative Analysis

Metabolite quantification was performed using a HPLC with an Aminex HPX-87 H column (BioRad Laboratories) coupled to a refractive index detector. The operation conditions were set as follows: 5 mM H_2_SO_4_ solution, column temperature 65 °C, eluent flowrate 0.60 mLmin^− 1^, and 0.02 mL injection volume.

## Results and Discussion

### Growth of *S. cerevisiae* Strain 202-3

*S. cerevisiae* yeast is a eukaryotic organism, classified as a microscopic fungus that grows as individual cells. Its doubling-time generation takes approximately 90 min and colonies containing millions of cells are produced after only 2 days of growth. It is recognized as a safe (Generally Recognized as Safe-GRAS) [9] and excellent sugar fermenter to produce ethanol, both in the presence and in the absence of oxygen. By itself, it tolerates well its own fermentation product (more than 15 % ethanol, in vv^− 1^), and withstands acidic conditions, temperature variations, and osmotic stress. It is the most used microorganism for production of beverage and food at industrial scale. It has a high resistance to the inhibitory compounds produced in lignocellulosic hydrolysates, thus being able to convert phenolic compounds and furaldehydes into less toxic compounds [10]. Nevertheless, according to the previous studies, this yeast cannot metabolize pentoses efficiently, such as the xylose present in the hydrolysates, since it has a low expression of the genes that code the xylose reductase (XR), xylitol dehydrogenase (XDH), and xylulose kinase (XK) enzymes, necessary for the metabolization of that pentose [11]. This is in agreement with the scientific dogma that *S. cerevisiae* cannot grow on xylose [12], although some old reports show that natural strains of this species can grow on that carbon source but modestly and very slowly [13, 14].

From our experiments, strain 202-3 emerges as a yeast possessing an innate ability to metabolize xylose, with the attractive characteristic of belonging to the genus and species *S. cerevisiae*. Although some studies report *S. cerevisiae* strains capable of fermenting xylose, most of them refer to genetically modified ones [15].

Cultures on the three solid media YNB minimal, YPX rich and Verduyn, using 20 gL^− 1^ of xylose as the sole carbon source, show that biomass formation indicates a positive phenotype in xylose assimilation, probably due to the presence of one or more enzymes involved in the metabolism of this pentose.

### Species Confirmation

With the primers used, described in the study of Muir, Harrison, and Wheals, 2011 [16] for *S. cerevisiae* ScerF2 (5′-GCGCTTTACATTCAGATCCCGAG-3′) and ScerR2 (5′TAAGTTGGTTGTCAGCAAGATTG-3′), and PCR analysis performed with the DNA from strain 202-3, a fragment of the expected size for the *S. cerevisiae* of 150 bp was obtained.

Using the primers ITS1 (5′-TCCGTAGGTGAACCTGCGG-3′) and ITS4 (5′-TCCTCCGCTTATTGATATGC-3′), a fragment of approximately 880 bp was obtained as expected for *S. cerevisiae* accordingly to [17]. The analysis of the sequencing results was performed by BLAST tool. The consensus sequence (GenBank OP104965) was constructed with the sequencing results of ITS1 and ITS4 and its result allowed concluding that the ITS region of the yeast strain 2023 belongs to *S. cerevisiae*, with a similarity greater than 99 %. Considering these and previous results, it was confirmed that strain 202-3 is indeed a *S. cerevisiae*.

### Physiological Characterization of 202-3 Strain Under Aerobic, Microaerobic, and Anaerobic Conditions

The consumption, growth, and fermentation measurements under various conditions of initial substrate concentration, oxygen availability, or medium types allowed establishing that strain 202-3 is a xylose positive “wild type” strain since it grows and transforms this pentose into xylitol.

In trials not shown, strain 202-3 showed excellent performance as a glucose fermenter with ethanol yields of 96.09 % under microaerobic or anaerobic conditions in YPD, and 91.40 % under microaerobic conditions in Verduyn. These yields are better than the reported in [18, 19] for industrial strains such as NAN-27 strain with ethanol yield of 82.39 %, 6508 strain with ethanol yield of 83.17 %, or PE-2 strain with ethanol yield of 92 ± (1.12) %. For the evaluated conditions, a complete glucose consumption was observed in less than 12 h and under anaerobic conditions an ethanol production yield of 0.491 gg^− 1^, close to the theoretical one of 0.511 gg^− 1^, was attained. Therefore, the 202-3 strain shows an excellent potential to obtain biofuels such as ethanol besides its inherent ability to metabolize xylose.

**Fig. 1:**
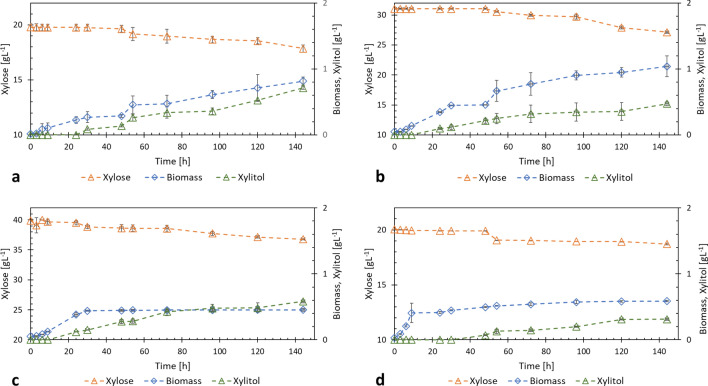
Consumption of xylose and production of xylitol by strain 202-3: **a** under microaerobic conditions in YPX medium 20 gL^− 1^, **b** under microaerobic conditions in YPX medium 30 gL^− 1^, **c** under microaerobic conditions in YPX medium 40 gL^− 1^ and **d** under anaerobic conditions in YPX medium 20 gL^− 1^

Microaerobic and anaerobic experiments with YPX medium showed that xylose consumption was greater for the microaerobic condition in (30 ± 1) gL^− 1^ of YPX, resulting in 12.6 % of consumption. The best xylitol yield was 0.368 gg^− 1^ for microaerobic condition in YPX 20 gL^− 1^. Figure 1 shows the xylose consumption and xylitol production by strain 202-3 under microaerobic conditions in YPX and Table 1 compiles the values of growth kinetic parameters of strain 202-3 under microaerobic and anaerobic conditions related to the consumption of xylose.

**Table 1:** Kinetic parameters of strain 202-3 in xylose under microaerobic and anaerobic conditions

202-3 strain	Y_X/S_	Y_Xyl/S_	P_X_	µ_max_
	[gg^− 1^]	[gg^− 1^]	[g L^− 1^ h^− 1^]	[h^− 1^]
Micro-YPX 20 gL^− 1^	0.412 ± 0.032	0.368 ± 0.016	0.006 ± 0.001	0.014 ± 0.004
Micro-YPX 30 gL^− 1^	0.253 ± 0.037	0.121 ± 0.009	0.007 ± 0.001	0.013 ± 0.001
Micro-YPX 40 gL^− 1^	0.129 ± 0.001	0.188 ± 0.014	0.003 ± 0.001	0.063 ± 0.001
Airlock-YPX 20 gL^− 1^	0.410 ± 0.007	0.230 ± 0.010	0.004 ± 0.001	0.005 ± 0.004

Despite the presence of glucose in the YPDX medium, strain 202-3 maintained xylose consumption, although it was reduced by 11.9 % compared to the YPX medium with only xylose as carbon source. This result suggests that the reduction in xylose consumption is due to the presence of glucose in the medium since *S. cerevisiae* prefers the consumption of this hexose.

In supplementary information is shown a figure (Figure S1) with the results of xylose and glucose consumption (a) under microaerobic conditions by strain 202-3 and (b) under anaerobic conditions in YPDX medium (20 gL^− 1^ glucose + 20 gL^− 1^ xylose). Also shown (Figure S1 (c)) is the growth of strain 202-3 in the Verduyn medium with 2 gL^− 1^ of glucose + 8 gL^1^ of xylose. Here, the small amount of glucose allowed a consumption of 27.7 % of the xylose in the first 14 h. After more than 200 h of growth experiment, only 33.22 % of the xylose had been consumed, i.e. an additional 5.5 % after the first 14 h. These results reveal an interesting behavior, since apparently the added amount of glucose allowed the strain to improve its uptake of xylose, by favoring the transport of this sugar into the cell. Xylose uptake in *S. cerevisiae* occurs by facilitated diffusion since it is transported by some hexose permeases with low affinity for pentose of the *HXT* gene family [20]. This suggests that the small amount of glucose enabled the overexpression of some of these genes, thus allowing xylose to enter the interior of the cell more easily in the first hours of the experiment.

**Fig. 2:**
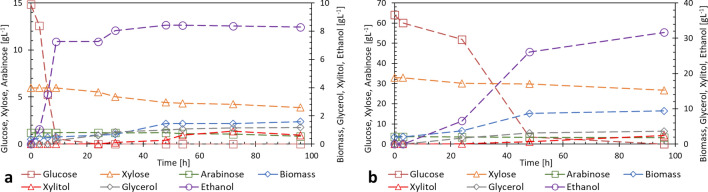
Fermentation of strain 202-3 in Hydrolysates-a **a** glucose 15 gL^− 1^, xylose 6 gL^− 1^, arabinose 1.2 gL^− 1^ and Hydrolysates-b **b** glucose 64 gL^− 1^, xylose 33 gL^− 1^, arabinose 3.7 gL^− 1^

Since it is desirable to take advantage of as much of the lignocellulosic biomass as possible, continuous research is being conducted to find microorganisms capable of carrying out a complete fermentation of all the sugars present in hemicellulosic hydrolysates and also that have a good tolerance to the presence of inhibitors produced in the lignocellulose pretreatment process. In the two fermentations on sugar cane bagasse hydrolysates with 202-3 strain the glucose was completely consumed for Hydrolysate-a in less than 12 h and a xylose consumption of 35 % and an arabinose consumption of 11 % were observed at the completion of 96 h as shown in Fig. 2(a), despite the presence of inhibitors such as acetic acid (3 gL^− 1^) and hydroxymethylfurfural (0.05 gL^− 1^). For Hydrolysate-b, Fig. 2(b) shows a complete consumption of glucose at 96 h with xylose consumption of 18.9 % and arabinose consumption of 13 %. The presence of acetic acid (7.5 gL^− 1^), hydroxymethylfurfural (0.2 gL^− 1^) and furfural (0.9 gL^− 1^) was measured in the initial hydrolysate. These values had no significant variation during the development of the experiment. The observed phenotype of 202-3 is an interesting and desirable condition to the efficient conversion of lignocellulosic biomass into fuels and chemicals products such as xylitol and arabinol.

For strain 202-3, fermentations with the Hydrolysate-b showed ethanol production with a yield of 0.513 g _ethanol‧_ g^− 1^_sugar_, even better than other industrial strains such as PE-2 that used corn stover hydrolysates with a concentration of 8.23 gL^1^ of glucose obtaining a yield of 0.38 g _ethanol‧_ g^− 1^_sugar_ [21].

Moreover, it was possible to observe the xylose consumption by the strain and the xylitol production under the conditions used in a fully defined Verduyn medium at a concentration of 10 gL^− 1^ of sugar. In the Supplementary Information, Figure S2 shows the results of the growth of strain 202-3 in Verduyn medium using xylose as the sole carbon source.

According to the results obtained in this experiment with Verduyn medium and with previous experiments with YPX medium, the highest xylitol production was obtained under microaerobic conditions, although the oxygen supply was not measured. It is well known that oxygen plays an important role in xylitol production by yeast being favored under microaerobic conditions. [22], which could be explained by three main reasons. First, in the yeast pentose phosphate pathway, the enzyme xylose reductase XR dependent on the cofactor NADPH reduces xylose to xylitol. Second, the enzyme xylitol dehydrogenase XDH dependent on the cofactor NAD^+^ oxidizes xylitol to convert it to xylulose. Third, a redox imbalance is generated between these cofactors, allowing the accumulation of the xylitol metabolite if the NAD^+^ regeneration is inefficient, since both cofactors have different oxygen requirements.

Studies on *Candida magnoliae* have shown that microaerobic conditions are advantageous for xylitol production, reaching a yield of 0.75 gg^− 1^, corresponding to 82 % of the theoretical value [23]. The xylitol yield reached for 202-3 strain in microaerobic condition in Verduyn medium was 0.25 gg^− 1^. The result obtained in this test may be related to the fact that an accumulation of NADP^+^ and NADH occurs under microaerobic conditions [24] which generates the intracellular redox imbalance. Therefore, it can be suggested that microaerobic conditions favor the NADPH regeneration in the pentose phosphate pathway, thus increasing xylitol production.

Although it is commonly accepted that the yeast *S. cerevisiae* is unable to assimilate xylose, early reports showed that several *Saccharomyces* strains have indeed a modest xylose consumption with very low rate as are presented in Supplementary information contained in the online version (see Table with data from this work and from 5, 14, 25), in which are listed just the xylose consumption in mediums with that pentose. However, 202-3 strain shows a better xylose consumption than the other reported wild strains and greater xylitol production. Moreover, we extend the study by also performing fermentations on hemicellulose hydrolysates in the presence of inhibitors to observe the fermentative potential of this wild strain obtaining from natural habitats.

## Conclusion

The wild yeast strain 202-3 isolated in Colombia, confirmed to belong to the species *Saccharomyces cerevisiae* using specific primers and by ITS region sequencing, shows xylose sugar metabolization into xylitol production. The xylose transformation was verified by means of different tests in which the only carbon source was that pentose. Despite the relatively slow and incomplete xylose consumption, this natural strain 202-3 metabolize pentoses such as xylose and arabinose in hemicellulose hydrolysates in presence of inhibitors which was not reported until now, besides an excellent yield of ethanol from glucose consumption. For the different tests performed with xylose as the sole carbon source, the best condition that favored xylitol production with strain 202-3 was the microaerobic condition. Our results confirm xylose consumption and xylitol production and suggest that through genetic and evolutive engineering this characteristic can be improved. Other natural strains of *S. cerevisiae* can be potential objects of study in the search for xylose-fermenting yeast and deserved to be analyzed in future studies.

## Electronic Supplementary Material

Below is the link to the electronic supplementary material.

Supplementary Material 1

## References

[1] Howard RL, Abotsi E, van Rensburg  EJ (2003). Lignocellulose biotechnology: issues of bioconversion and enzyme production. Afr J Biotechnol.

[2] Patel SKS, Das D, Kim SC (2021). Integrating strategies for sustainable conversion of waste biomass into dark-fermentative hydrogen and value-added products. Renew Sustain Energy Rev.

[3] Cifuentes Y, Latorre S, Pinzón A et al (2015) In: IEEE 5th International Conference on Computational Advances in Bio and Medical Sciences (ICCABS), Oct. 2015, pp. 1–2. 10.1109/ICCABS.2015.7344727

[4] Patiño MA, Ortiz JP, Velásquez M (2019). D-Xylose consumption by nonrecombinant *Saccharomyces cerevisiae*: a review. Yeast.

[5] Gong CS, Claypool TA, McCracken LD (1983). Conversion of pentoses by yeasts. Biotechnol Bioeng.

[6] Hsiao HY, Chiang LC, Ueng PP (1982). Sequential utilization of mixed monosaccharides by yeasts. Appl Environ Microbiol.

[7] VanZyl CV, Prior BA, Kilian SG et al (1989) D-xylose utilization by *Saccharomyces cerevisiae.* J Gen Microbiol 135(11):2791–2798.10.1099/00221287-135-11-2791 10.1099/00221287-135-11-2791 2515242

[8] Lancheros-Castaneda S, Fonseca DM, Lozano MV (2015). Increase in second generation ethanol production by different nutritional conditions from sugarcane bagasse hydrolysate using a *Saccharomyces cerevisiae* native strain. Chem Eng Trans.

[9] Turner TL, Kim H, Kong II (2016). Engineering and evolution of *Saccharomyces cerevisiae* to produce biofuels and chemicals. Adv Biochem Eng Biotechnol.

[10] Jönsson LJ, Alriksson B, Nilvebrant N-O (2013). Bioconversion of lignocellulose: inhibitors and detoxification. Biotechnol Biofuels.

[11] Sato TK (2016). Directed evolution reveals unexpected epistatic interactions that alter metabolic regulation and enable anaerobic xylose use by *Saccharomyces cerevisiae*. PLoS Genet.

[12] Hou J, Qiu C, Shen Y (2017). Engineering of *Saccharomyces cerevisiae* for the efficient co-utilization of glucose and xylose. FEMS Yeast Res.

[13] Attfield PV, Bell PJL (2006). Use of population genetics to derive nonrecombinant *Saccharomyces cerevisiae* strains that grow using xylose as a sole carbon source. FEMS Yeast Res.

[14] Wenger JW, Schwartz K, Sherlock G (2010). Bulk segregant analysis by high-throughput sequencing reveals a novel xylose utilization gene from *Saccharomyces cerevisiae*. PLoS Genet.

[15] Matsushika A, Goshima T, Hoshino T (2014). Transcription analysis of recombinant industrial and laboratory *Saccharomyces cerevisiae* strains reveals the molecular basis for fermentation of glucose and xylose. Microb Cell Factories.

[16] Muir A, Harrison E, Wheals A (2011). A multiplex set of species-specific primers for rapid identification of members of the genus *Saccharomyces*. FEMS Yeast Res.

[17] Korabecna M (2007) The variability in the fungal ribosomal DNA (ITS1, ITS2, and 5.8 S rRNA Gene): its biological meaning and application in medical mycology,. In: Communicating current research and educational topics and trends in applied microbiology, Méndez-Vilas

[18] Li H (2015). Evaluation of industrial *Saccharomyces cerevisiae* strains as the chassis cell for second-generation bioethanol production. Microb Biotechnol.

[19] Basso LC, de Amorim HV, de Oliveira AJ (2008). Yeast selection for fuel ethanol production in Brazil. FEMS Yeast Res.

[20] Stambuk BU, Eleutherio ECA, Florez-Pardo LM et al (2008) “Brazilian potential for biomass ethanol: challenge of using hexose and pentose co-fermenting yeast strains,” pp.918–926,

[21] Bohn LR (2021). Alkaline pretreatment and enzymatic hydrolysis of corn stover for bioethanol production. Res Soc Dev.

[22] Morais CG, Cadete RM, Uetanabaro APT (2013). D-xylose-fermenting and xylanase-producing yeast species from rotting wood of two Atlantic Rainforest habitats in Brazil. Fungal Genet Biol.

[23] Nakano K, Katsu R, Tada K (2000). Production of highly concentrated xylitol by *Candida magnoliae* under a microaerobic condition maintained by simple fuzzy control. J Biosci Bioeng.

[24] Bruinenberg PM, de Bot PHM, Dijken J (1984). NADH-linked aldose reductase: the key to anaerobic alcoholic fermentation of xylose by yeasts. Appl Microbiol Biotechnol.

[25] Batt CA, Carvallo S, Easson DD (1986). Direct evidence for a xylose metabolic pathway in *Saccharomyces cerevisiae*. Biotechnol Bioeng.

