# Supplementary material for: Xylose Metabolization by a Saccharomyces cerevisiae Strain Isolated in Colombia
Source: Indian J Microbiol. 2023 Feb 18;63(1):84–90. doi: 10.1007/s12088-023-01054-z (PMC10172406; doi:10.1007/s12088-023-01054-z)
Supplement: Supplementary file 1 — Supplementary Material 1 [file 12088_2023_1054_MOESM1_ESM.docx]

**Xylose metabolization by a *Saccharomyces cerevisiae* strain isolated in Colombia**

**Supplementary information**


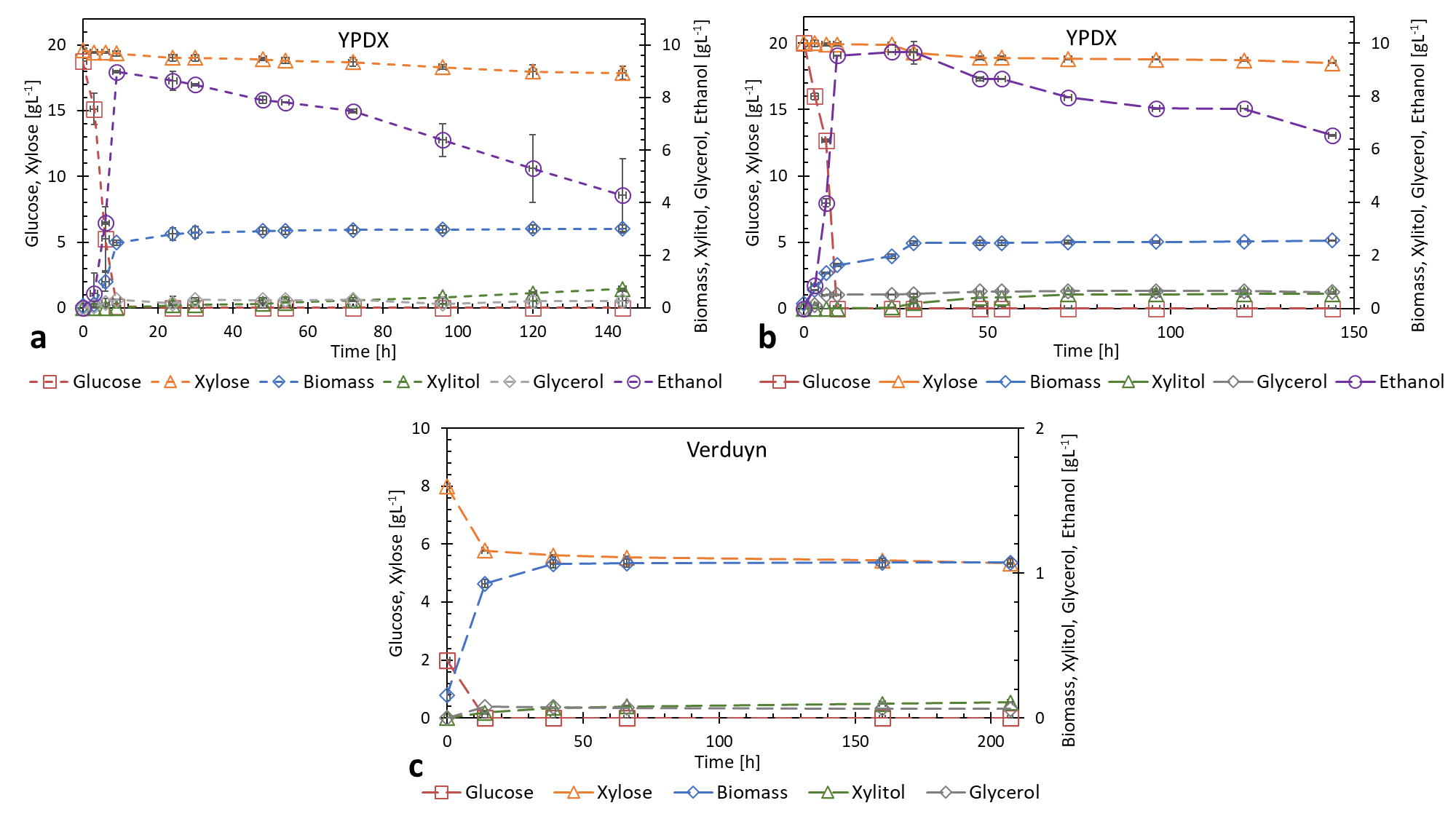


Figure S1. Glucose+xylose consumption by strain 202-3 in YPDX medium 20 gL^-1^ glucose+20 gL^-1^ xylose: (a) under microaerobic and (b) anaerobic conditions. (c) Growth in Verduyn medium (2 gL^-1^ glucose+8 gL^-1^ xylose).


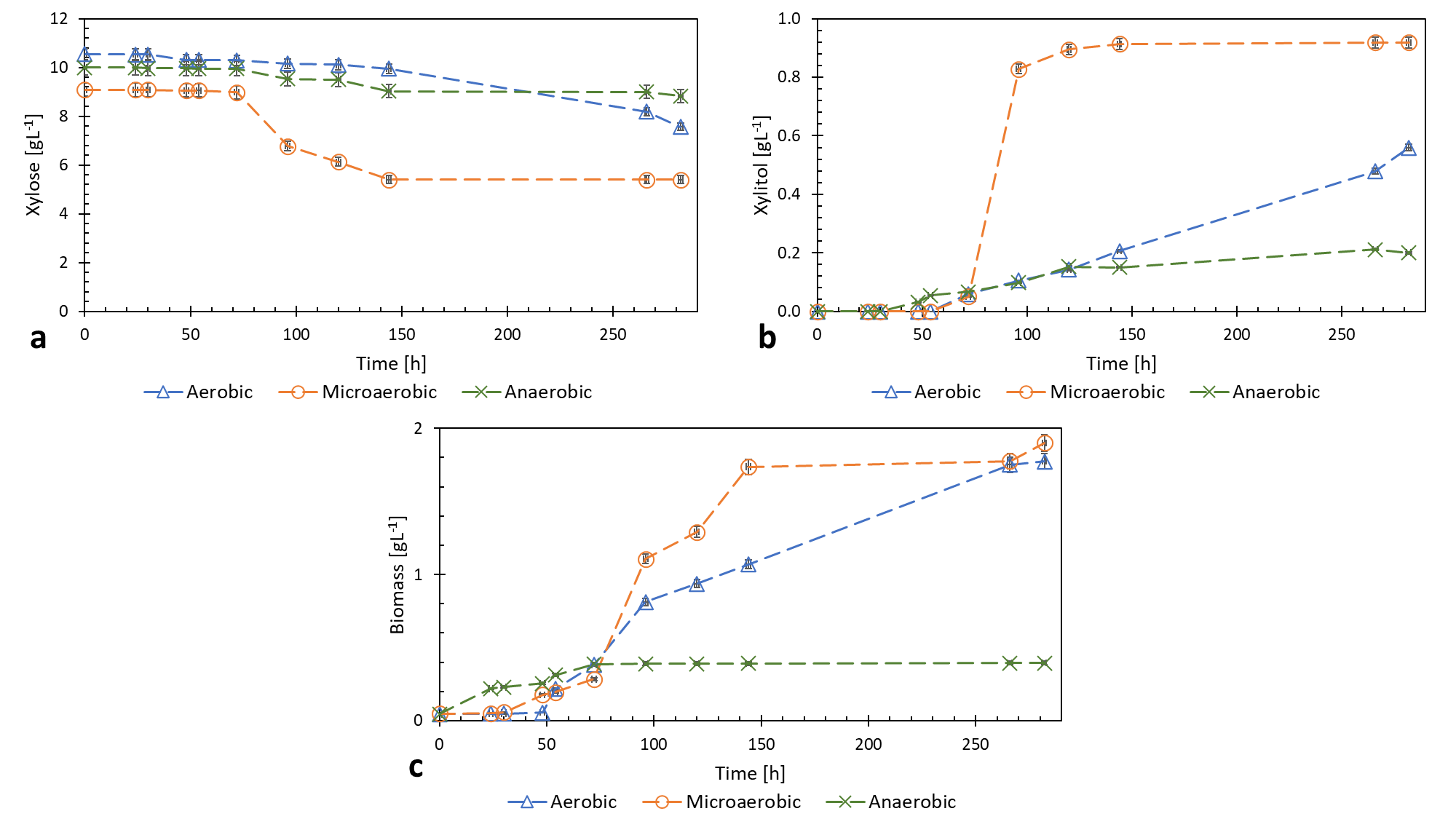


Figure S2. Growth of strain 202-3 in Verduyn medium of 10 gL^-1^ of xylose, under aerobic, anaerobic and microaerobic conditions: comparison of (a) xylose consumption, (b) xylitol production and (c) biomass production.

**Supplementary Table.** Reported strains of *Saccharomyces cerevisiae* with xylose consumption or growth in that pentose.

| *S. cerevisiae* strain | Conditions | Xylose consumption or growth [gL^-1^] | Xylitol production | Reference |
| --- | --- | --- | --- | --- |
| 202-3 | Microaerobic conditions  (20 gL^-1^ xylose) | 1.94 | 0.71 | This study |
| 202-3 | Microaerobic conditions  (30 gL^-1^ xylose) | 3.91 | 0.47 | This study |
| 202-3 | Microaerobic conditions  (40 gL^-1^ xylose) | 3.11 | 0.59 | This study |
| 202-3 | Anaerobic conditions  (20 gL^-1^ xylose) | 1.36 | 0.31 | This study |
| ATCC 4126 | Fermentative conditions  (50 gL^-1^ xylose) | 0.59 | 0.16 | Gong *et al.,* 1983 [5] |
| ATCC 4132 | Fermentative conditions  (50 gL^-1^ xylose) | 0.36 | 0.08 | Gong *et al*., 1983 [5] |
| ATCC 9763 | Fermentative conditions  (50 gL^-1^ xylose) | 0.42 | 0.10 | Gong *et al.,* 1983 [5] |
| ATCC 24553 | Fermentative conditions  (50 gL^-1^ xylose) | 0.44 | 0.13 | Gong *et al.,* 1983 [5] |
| ATCC 24857 | Fermentative conditions  (50 gL^-1^ xylose) | 0.68 | 0.26 | Gong *et al.,* 1983 [5] |
| ATCC 24859 | Fermentative conditions  (50 gL^-1^ xylose) | 0.66 | 0.42 | Gong *et al.,* 1983 [5] |
| ATCC 26497 | Fermentative conditions  (50 gL^-1^ xylose) | 0.66 | 0.26 | Gong *et al.,* 1983 [5] |
| ATCC 26497 | Aerobic conditions  (50 gL^-1^ xylose) | 1.63 | 0.34 | Gong *et al.,* 1983 [5] |
| ATCC 26603 | Aerobic conditions  (50 gL^-1^ xylose) | 0.52 | 0.09 | Gong *et al.,* 1983 [5] |
| ATCC 26785 | Fermentative conditions  (50 gL^-1^ xylose) | 0.64 | 0.29 | Gong *et al.,* 1983 [5] |
| A364A | Aerobic conditions | Positive growth DO_600 nm_ | Unanalyzed | Batt *et al.,* 1986  [25] |
| Montrachet | Aerobic conditions | Positive growth DO_595 nm_* | Unanalyzed | Wenger *et al.,* 2010 [14] |
| Premier Cuvee | Aerobic conditions | Positive growth DO_595 nm_* | Unanalyzed | Wenger *et al.,* 2010 [14] |
| UCD819 | Aerobic conditions | Positive growth DO_595 nm_* | Unanalyzed | Wenger *et al.,* 2010 [14] |
| G30 #2 | Aerobic conditions | Positive growth DO_595 nm_* | Unanalyzed | Wenger *et al.,* 2010 [14] |
| 921 PRF21-2 | Aerobic conditions | Positive growth DO_595 nm_* | Unanalyzed | Wenger *et al.,* 2010 [14] |
| SIHA Activ-hefe 4 | Aerobic conditions | Positive growth DO_595 nm_* | Unanalyzed | Wenger *et al.,* 2010 [14] |
| Fermichamp | Aerobic conditions | Positive growth DO_595 nm_* | Unanalyzed | Wenger *et al.,* 2010 [14] |
| BP725 | Aerobic conditions | Positive growth DO_595 nm_* | Unanalyzed | Wenger *et al.,* 2010 [14] |
| Actiflore C (F33) | Aerobic conditions | Positive growth DO_595 nm_* | Unanalyzed | Wenger *et al.,* 2010 [14] |
| Lalvin AC | Aerobic conditions | Positive growth DO_595 nm_* | Unanalyzed | Wenger *et al.,* 2010 [14] |
| YJM270 | Aerobic conditions | Positive growth DO_595 nm_* | Unanalyzed | Wenger *et al.,* 2010 [14] |
| ATCC66283 | Aerobic conditions | Positive growth DO_595 nm_* | Unanalyzed | Wenger *et al.,* 2010 [14] |
| BDX | Aerobic conditions | Positive growth DO_595 nm_* | Unanalyzed | Wenger *et al.,* 2010 [14] |
| EC1118 | Aerobic conditions | Positive growth DO_595 nm_* | Unanalyzed | Wenger *et al.,* 2010 [14] |
| FA1 | Aerobic conditions | Positive growth DO_595 nm_* | Unanalyzed | Wenger *et al.,* 2010 [14] |
| French White | Aerobic conditions | Positive growth DO_595 nm_* | Unanalyzed | Wenger *et al.,* 2010 [14] |
| Premier Cuvee | Aerobic conditions | Positive growth DO_595 nm_* | Unanalyzed | Wenger *et al.,* 2010 [14] |
| Simi White | Aerobic conditions | Positive growth DO_595 nm_* | Unanalyzed | Wenger *et al.,* 2010 [14] |
| CS2 | Aerobic conditions | Positive growth DO_595 nm_* | Unanalyzed | Wenger *et al.,* 2010 [14] |
| SIHA Activ-hefe 3 | Aerobic conditions | Positive growth DO_595 nm_* | Unanalyzed | Wenger *et al.,* 2010 [14] |
| 71B | Aerobic conditions | Positive growth DO_595 nm_* | Unanalyzed | Wenger *et al.,* 2010 [14] |
| PDM | Aerobic conditions | Positive growth DO_595 nm_* | Unanalyzed | Wenger *et al.,* 2010 [14] |
| Primeur | Aerobic conditions | Positive growth DO_595 nm_* | Unanalyzed | Wenger *et al.,* 2010 [14] |
| Enoferm M1 | Aerobic conditions | Positive growth DO_595 nm_* | Unanalyzed | Wenger *et al.,* 2010 [14] |
| Fermicru LVCB | Aerobic conditions | Positive growth DO_595 nm_* | Unanalyzed | Wenger *et al.,* 2010 [14] |
| WE14 | Aerobic conditions | Positive growth DO_595 nm_* | Unanalyzed | Wenger *et al.,* 2010 [14] |

* Positive growth DO_595 nm_ TECAN Genios plate reader with orbital shaking.
